# Ageing and Elderly Care in the Arab Region: Policy Challenges and Opportunities

**DOI:** 10.1007/s12126-016-9244-8

**Published:** 2016-04-06

**Authors:** Shereen Hussein, Mohamed Ismail

**Affiliations:** 1 Social Care Workforce Research Unit, King’s College London, Strand, London, WC2R 2LS UK; 2 Analytical Research Ltd, Station House, Connaught Road, Surrey, GU24 0ER UK

**Keywords:** Long-term care, Policy, Women multiple roles, Women, Ageing, Middle East, North Africa

## Abstract

Populations are expected to age rapidly in the Arab countries during the coming few decades. However, the current evidence base indicates that many countries in the region are not paying attention to this demographic phenomenon. This is a particular concern as longevity is often accompanied by many years of ill health and disability and most of the countries in the region continue to rely on the family as the primary source of elder care. While the family, and particularly women, are expected to provide increasing support for longer, they are faced by a set of socio-demographic changes that may hinder their ability to provide such care. This paper focuses on the ageing demographics in the Arab region and reflects on the multiple-roles for women by utilising quantitative analysis of international population and socio-economic indicators as well as reviewing the background literature and current ageing policies in the region. The paper then discusses possible strategies to address increasing long-term care needs through a social capital lens, where support to informal carers particularly women is emphasised.

## Introduction

As in many other parts of the world, most Arab countries^i^ are experiencing demographic transitions including lower fertility, lower mortality and longer life expectancy. The population structure of the Arab region is still young, with nearly half of the population younger than the age of 25 in 2009; which is compared to only 29 % in developed countries (United Nations [UN] 2009). However, the demographic transition of reduced fertility and mortality rates has accelerated the process of population ageing in the region. While many of the Arab countries are not currently experiencing population ageing at the same level as most developed countries, the majority of the countries are in the cusp of predicted significant demographic changes particularly in relation to the pace of population ageing. By 2050, the proportion of older persons (60 years or more) is predicted to climb to 19 % compared to an average of around 7 % in 2010 (UN 2013). Because of historical high fertility rates, the number of older persons is predicted to more than quadruple from 22 million in 2010 to 103 million by 2050. In nine countries in the region - Algeria, Bahrain, Kuwait, Lebanon, Libya, Morocco, Oman, Qatar and Tunisia - there will be more older persons than children (under 15 years old) by 2050 (UN 2013).

Most people in the Arab countries share similar historical and cultural backgrounds, founded in the main around religion, yet they are not a homogenous group. The cultural and political status of women, their fertility levels as well as the laws governing family and marriage, vary widely across the region. Levels of life expectancy in many Arab countries is a great success story of social and economic development, however, it is also one of the most profound public policy challenges of the 21st century. This is particularly the case for many countries in the region, which appear not to be equipped to address the multiple implications of such changes (Kronfol et al. 2013). Population ageing in the Arab world has been occurring in a context of parallel and major socio-economic and socio-political changes challenging traditional family structure norms and intergenerational support systems. Furthermore, the implications of rapid ageing in several Arab countries are often not acknowledged by policy makers, with research showing that public welfare and policy or strategies to address population change remains limited albeit gaining some recent attention (Sibai and Yamout 2012; Yount 2005).

The tempo, or speed, of the population ageing process has been different for some countries in the region, with some identified as having ‘fast’, others as ‘medium’ and ‘slow’ tempos (Saxena 2008). Within the ‘fast’ or rapidly ageing group are the United Arab Emirates, Tunisia, Bahrain, Kuwait, Morocco, Algeria, Bahrain, Libya and Lebanon (Saxena 2008). These countries are also experiencing epidemiological and health transitions, with non-communicable diseases replacing communicable diseases as the leading causes of morbidity and mortality (Abyad 2006).

In spite of the above, research examining care for the elderly in the Arab world, including ill health in later life, long-term care provision, and the use of formal or paid care services remains surprisingly sparse (Sibai and Yamout 2012; Yount and Sibai 2009). Although older people in the region have traditionally tended to live with or near their offspring, larger proportions of the, notably older women, are increasingly living alone (Sibai et al. 2009). This situation is exacerbated by both internal and international migration patterns, where younger men are more likely to migrate leaving their wives, usually with young children and older parents to care for, with multiple responsibilities (Fargues 2006). Concurrently, the viability of informal, traditional forms of long-term care in the family unit are threatened due to various factors such as modernization, urbanization and youth migration.

A key question remains however, how can Arab countries devise better elderly care policies that are suited to the new family structures that have emerged in the region which increasingly point to the unsustainability of an aged-care model centred around female kin? Addressing this important policy question will have important implications for several of the post-2015 Sustainable Development agenda goals, namely Goals 3 and 11: “Ensure healthy lives and promote wellbeing for all at all ages” and “Make cities and human settlements inclusive, safe, resilient and sustainable” (UN 2014).

## Aims and Methods

The aim of this paper is twofold: (1) to review the current socio-demographic position, in relation to ageing, of the Arab region reflecting on current model of care provision and policy strategies in the region; (2) to propose a new model of care and new sets of policies for elderly care which moves beyond the over-reliance on women and addresses the new realities posed by changing family and demographic structures in the Arab region. To this end, the paper draws on key policies and strategies that can be adopted to meet the growing multiple roles placed on women as providers of growing long term care needs in Arab region.

The paper starts with an overview of demographic and ageing patterns in the region in relation to other socio demographic indicators, such as female labour participation and literacy rates. This will provide the background discussion for the policy analysis that will follow, focusing on current ageing policy initiatives adopted by some countries in the region. The discussion will pay special attention to the unsustainable burden on women in informal long-term care provision. The paper then reflects upon the potential for new models of long-term care support in the Arab region both by reflecting on examples of elderly care provision that have been adopted by governments in more developed countries and by considering the specific cultural context of the Arab region.

The data sources for the analysis presented here are both qualitative and quantitative based on development and demographic databases published by various international bodies such as the World Bank and the United Nations. In addition, the authors make use of a systematic literature review of the role of women in long-term care in the Arab region. This entailed extensive searches of Several social science databases including AgeInfo, the Social Science Research Network (SSRN), Applied Social Science Index and Abstracts (ASSIA); Health Management Information Consortium Database (HMIC); Social Care Online (SCO); and Sociological Abstracts and Social Services Abstracts (SSA/SA) covering the period from 1995 to 2014. Systematic search terms were used to extract articles related to long-term care, informal care, and ageing/aging and multiple roles of informal caregivers, particularly women.

## Key Patterns of Demographic Changes in the Arab Region

The Arab region is characterized by demographic, socioeconomic and political diversity. Some countries such as Egypt have very large populations, 84 millions, while others such as Qatar have much smaller population of only 110,000 inhabitants. In the majority of countries, despite an observed ‘Youth Bulge’, population ageing is also occurring (Roudi-Fahimi and Kent 2007). This is indicated by increased life expectancy, taking place in a context of socio-economic change, and accompanied by fertility reduction and significant changes in family structure. For example, the proportion of people aged 60 or more (traditional pension age in most Arab countries) represents 6 % of total population in 2010. In some countries, such as Lebanon and Tunisia, older people constitute at least 10 % of the population. More importantly, the projected relative representation of this group is expected to go up to 19 % by 2050. In some countries, such as Egypt, due to its large population of over 84 millions, this equates to a considerable number of older people and prompts for policy and practice consideration.

While not all longevity is associated with ill health and increased need for care provision, the proportion of the population that is living longer means that more people will need support and care as they move to end of life (Boggatz and Dassen 2005; Hussein et al. 2009; Sibai et al. 2004; Sinunu et al. 2009; Alzheimer Disease International 2011). Additionally, there is an evident gender-gap in the prevalence of widowhood in the region, with women more likely to be living alone in old age (Sibai et al. 2009). This is not only because of higher women average life-expectancy than that of men but also due to differences in gender-related marriage patterns. For example, relatively large wife-husband age gap, polygyny and lower remarriage rate for women following divorce or widowhood can also be explanatory factors for larger proportions of women living alone at old age (Hussein 2002; Hussein and Manthorpe 2007; Yount and Sibai 2009). For example, 85 % of men aged 60 or more are married compared to only 39 % of women in the same age group in North Africa (UN 2009). These factors combined with socio-demographic changes impact on both intergenerational linkages and living arrangements among older women including solitary living at old-age (Shah et al. 2002; Yount and Khadr 2008).

**Fig. 1:**
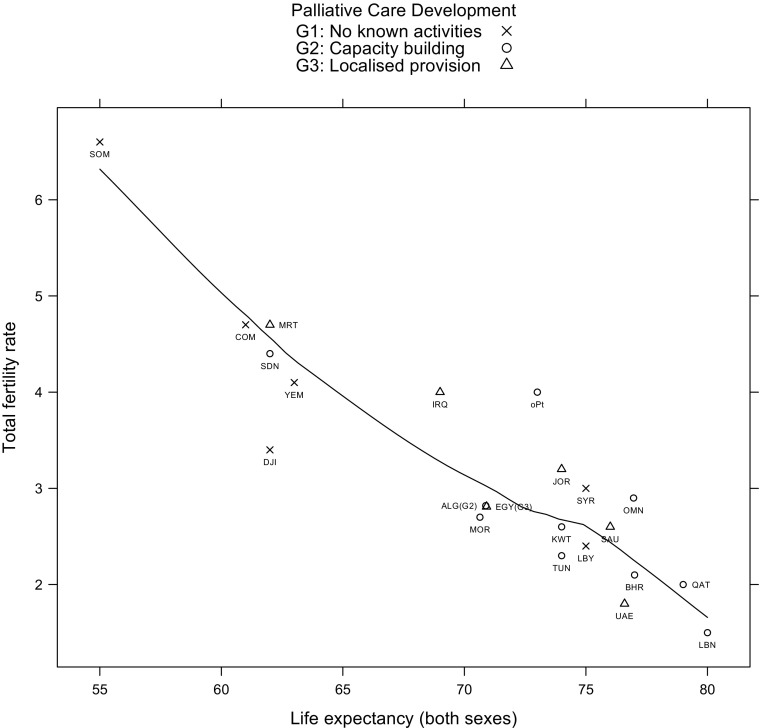
Total fertility rate by life expectancy in the 22 Arab countries grouped by level of palliative care development Source: Life expectancy and total fertility rate refer to rates in 2013 World Bank http://data.worldbank.org/indicator/SP.DYN.LE00.MA.IN

Figure 1 presents the distribution of Arab countries by life expectancy and fertility rates. The countries are also grouped according to their levels of palliative care provision according to the typology developed by Clark and Wright (2007). Palliative care is defined as provision of end of life care and is used here as an indicator of a country’s recognition of their population ageing, palliative care could be provided by government, private, non-governmental or charitable organisations. Clark and Wright (2007) developed a world map with four main groups of countries: 1) no known palliative care activity, 2) countries with palliative care capacity building activity, 3) countries with localized provision of palliative care, and 4) countries where palliative care activities are approaching integration with the wider public health system. None of the Arab countries were identified by Clark and Wright (2007) to belong to Group 4.

Figure 1 clearly shows the diversity of the Arab countries in relation to their position at the demographic transition. At one end we find Somalia still exhibits very high total fertility rate (6.6) and low average life expectancy (55 years). At the other end, Lebanon is almost singled out with the lowest total fertility rate (1.8) and highest average life expectancy in the region (80 years). A group of countries - Comoros, Djibouti, Mauritania and Yemen - is clustered with medium range of fertility rates (between 3.4 and 4.7) and relatively low life expectancy (between 61 and 63). It is worth noting that the latter group has relatively low health expenditure per capita (ranging from 44$ to 137$; WHO 2013) and low human development index^ii^ (ranging from 0.47 to 0.5; UNDP 2013). Another larger group of countries – Bahrain, Jordon, Kuwait, Libya, Oman, Tunisia, Saudi Arabia, Syria and United Arab Emirated (UAE) - is situated at a latter phase of their second demographic transition, with relatively lower fertility rates (from 1.8 to 3.2) and higher life expectancy of between 74 and 77 years. Some countries appear to be on their way to reach the position of the latter group, such as Egypt, Algeria and Morocco; while Qatar appear to be in a near position to Lebanon in terms of fertility and life expectancy levels. When we consider these countries in relation to their palliative care provision alongside their demographic transition, we find that the relationship is not always consistent. For example, while Mauritania is at an early stage of demographic transition, it  has localised provision of palliative care (the most advanced group within the region) While Libya with relatively low fertility rate and high life expectancy has no known activities for palliative care. Lebanon while displaying the most advanced demographic indicators is still in the capacity building stage. On the other hand UAE appears to be paying attention to palliative care provision inline with the changes in their demographic changes.

Research available on the Arab region indicates that a considerable part of the increased life expectancy in this region is spent in ill-health. For example, Margolis and Reed (2001) found that older people using formal care services in the UAE had higher rates of neurological diseases and/or dementia than in Western countries, such as the United States (US). Similar findings from Iran were illustrated by Tajvar et al. (2008), where they highlight considerable poor health indicators among older women.

## Key Trends in Care Support for the Elderly in the Arab Region

There are two main (often parallel) systems of long-term care for the elderly in the Arab region: informal care providers, such as unpaid family members, as well as formal care providers, such as nursing aides, home care assistants, and other paid care workers. Most care delivered to older people, or people with disabilities or long-term care needs, in the Arab region is provided by family members, mainly women, or by other informal caregivers (Hoodfar 1997; Rugh 1984, 1997; Sibai et al. 2012; Yount and Rashad 2008). Long term care in the majority of cases is family-based, mainly because of deeply-rooted religious and cultural norms that emphasis the duties of younger generations towards their elders, but also due to critical lack of formal care alternatives (as might be provided by the public policy systems or the private sector). Indeed, where private care is provided such as through a private nursing home or a non-governmental organization specializing in social care, there is much social stigma attached to the idea of elderly relatives being placed in such nursing homes. Extended hospitalizations and the admission of frail, older people to care facilities have occurred in many Arab countries for at least the past two to three decades, these were usually attributed to combined effects of longevity and lack of other suitable formal care provisions that is home or community-based (Rugh 1981; Rugh 1984; Sibai et al. 2009; Sinunu et al. 2009). Indications of increased use of formal care, especially among urban older people are emerging. For example, research in Egypt (Boggatz and Dassen 2005; Sinunu et al. 2009) indicates that many factors are prompting family caregivers in Cairo to employ formal or paid care-workers to look after their older relatives, despite the long-standing norms of family care. However, there are no statistics available to establish the volume of or trends in the use of formal or paid care, mainly because such arrangements are often poorly documented (Margolis and Reed 2001).

Nevertheless, the continued availability or indeed willingness of family members to care for elderly relatives is uncertain, due to a number of interacting demographic and socioeconomic trends. In particular, changes in family structure and youth migration, combined with increases in women’s participation in the labour force, may negatively affect the availability and willingness of family members to provide care for their older relatives (Hussein and Manthorpe 2005). Additionally, many informal carers might not be equipped to the type of care associated with ageing, such as complex and dementia needs.

**Fig. 2:**
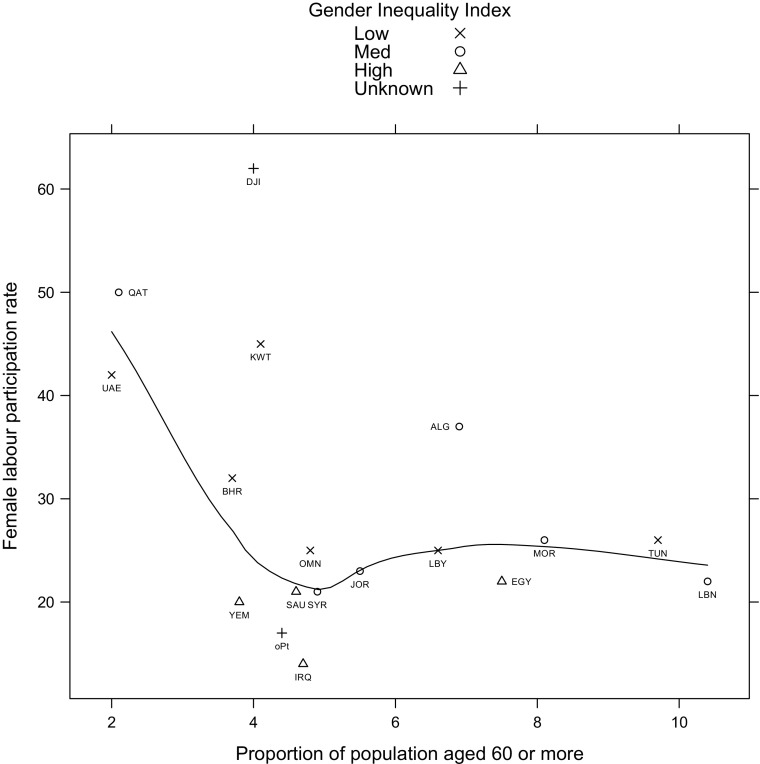
Female labour participation rate and proportion of the population aged 60 or more in the 22 Arab countries grouped by their relative level of Gender Inequality Index

Figure 2 presents the distribution of the 22 Arab countries by female labour participation rate (2011) and proportion of their population over the age of 60. The countries are then grouped according to their Gender Inequality Index (GII).^iii^ The GII is distributed into three groups based on the percentile distribution of all Arab countries; thus the levels of low, medium, high are relative to the rest of the ‘Arab’ countries and not the world distribution of GII. The higher the GII value the more disparities between females and males.

Figure 2 reflects the relatively young populations of the Gulf region, with high levels of female labour participation rates. However, it should be noted that the latter figures reflect the experience of migrant as well as native women in relation to labour market participation and thus may not provide an accurate indication of the competing roles of women who are expected to provide, or in some cases manage, elder care. Documented female labour participation rates are also likely to underrepresent informal and undocumented labour sectors, where women in the region are particularly concentrated (Jütting et al. 2008). Nevertheless, the data provides some indication of the position of different countries in relation to the three inter-related factors of ageing, multiple demands on women through labour participation and gender inequality. Egypt presents a situation where female participation rate is over 20 %, proportion of older people relatively high at around 8 % with relatively high GII; presenting a scenario of competing demands on women within unsupportive gender-sensitive policies. Tunisia presents a scenario where female labour participation is within the medium range, high proportion of older people but considerably low GII indicating relatively higher levels of gender equality.

## Elderly Care and Old-age policies in the Arab region

The Arab region can be characterized as having primarily residual social welfare systems that rely heavily on family or community-based social support, especially for those members of the population who do not have access to welfare benefits that are based on a record of formal employment-based social insurance contributions. This reliance on kin and community-based support is especially prominent in the case of social care where the state exercises little legislative power over the family. This formal neglect of social care as a constituent part of social policy in the Arab region arises from a male-breadwinner approach to social policy which prioritizes the economic activity of the man/father and assumes that the mother or female kin will take responsibility for the care needs of the nuclear or extended family members.

Within this overarching framework, long term care needs associated with old age tend to be seen as a family rather than societal responsibility within the region, with co-residency, or shared households, usually employed as a means to meet the needs of both the young and old generations within the family (UN 2011; Yount and Sibai 2009). While the Arab countries include some of the world’s wealthiest states, wealth distribution across the population is far from uniform, with significant pockets of poverty. For example, Yemen and Sudan have witnessed large increases in their populations living under the poverty line since early 1990s and are classified as some of the least developed by the United Nations (Mirkin 2010). Most of the countries in the region have relatively young traditional social insurance programmes. These programmes offer old-age statutory pensions, with a typically higher uptake in urban areas. It is estimated that social security coverage extends to less than 25 % of the population (Economic and Social Commission For Western Asia [ESCWA] 2006), and the majority of older people rely excessively on savings and informal support, including financial, from family and charities. It is likely that older women are more affected by the limited social security coverage, given their continued over representation in undocumented labour, such as agricultural and small trade.

Overall, older people appear to be marginalized in the health and welfare policy-making process in the region (Sibai et al. 2004). While the rights to equality of older people in most Arab countries are entrenched in their constitutions (UNFPA 2012) and recognition of the issues related to aging within the region has gained momentum in recent years, however, wide gaps between ratifications and implementations of policies remain.

According to the United Nations (2008) most countries in the Arab region view population ageing to be a ‘minor’ concern. Nevertheless, some Governments have recognised the need to meet future challenges associated with expected population ageing. An ESCWA study (Kronfol et al. 2013) identified a number of countries where home care services for older people are available, however, these are mostly covered and initiated by civil society and often religious-based organizations, sometimes subsidized through public funds. For example, in Egypt, the ‘Regular Medical Caravans’ provide free medical consultation and services - including minor surgeries at homes in rural areas; Bahrain has ten government-sponsored mobile clinics. In Tunisia, the ‘Union of Social Solidarity’ offers mobile teams to providing free home-based health services for the elderly. Tunisia also provides specialized government-funded rehabilitation and physical therapy services to older persons for little or no fees. Home-based care in Kuwait is entirely free of charge and NGOs in Morocco provide free medication, medical consultation to older persons in need and support families and caregivers of older persons with Alzheimer’s disease. In Jordan, the private sector has recently expanded to include up to 53 companies registered at the Ministry of Health that provide home care for older persons. In Lebanon, there are 26 mobile clinics for older people living at home. Two countries, Lebanon and Palestine, mentioned the availability of “meals on wheels” services that cater to older people living alone.

## The Role of Women in Long-Term Care Provision

The current picture of family formation, encompassing marriage and fertility, is far from homogenous (Hussein 2002; Hussein and Manthorpe 2007; Rashad and Khadr 2002; Yount and Rashad 2008), and women’s position in society vary dramatically within the Arab region. For example, Yount and Rashad (2008) highlight the striking variations in family structure and women’s political and economic participation in Egypt, Tunisia and Iran. For older women variations in levels of qualifications, social exclusion, financial capital and geographical segregation pose different sets of challenges.

For all Arab countries, changing demographic structures, labour market dynamics, migration and systems of financing long-term care, all influence care provision. They are also influenced by shifts in marital and intergenerational power relations and support (Hussein and Manthorpe 2007; Yount 2005). As discussed earlier, policy attention to long-term care needs and preventative policies remain under-developed. This is perhaps associated with the deeply-rooted cultural and traditional norms that emphasize the role of the family in wellbeing - and particularly the role of women - in the care provision and support system (Sibai and Yamout 2012). While there are a number of large religious minority groups such as Christians and Jewish people in the region, the vast majority of the region populations are Muslims and Arabic-speaking. Islamic beliefs and traditions also provide the basis of social order in many Arab countries thereby informing the many of the prevailing intergenerational support systems in the region. Within such context, women are usually portrayed and expected to be the main source of emotional and care support. However, Islam also places a considerable duty of elderly care, particularly financially, on male sons (El-Ashi 2007). The same code of practice may explain the large involvement of older people in financial and domestic help to their off-spring, including child care, where an unspoken system of obligations and duties is in place (Sibai and Yamout 2012). Yet, it is the day to day and hands on care duties that are implicitly, and many times explicitly, placed on daughters or daughters in laws.

While such traditional values remain at the heart of the Arab society, the practicalities of women delivering elderly care, along with their traditional roles of wives and mothers and meeting other competing and increasingly expected duties of labour participation, can be challenging to say the least. With increasing life expectancy and societal changes there are considerable forces that calls for new policy and governmental support that addresses care needs of older people outside the family sphere.

The literature review indicates that informal care continues to play a central role in long-term care, and women provide most formal and informal care. The available data indicate that the use of formal care services, such as care homes or home care services, by older people is not substantial, but that it may be growing. The literature emphasizes the enduring and sizeable role played by women in relation to informal long-term care, which is the most common form of care provision within the region. Even when hospitalization is necessary, bedside or personal care, such as help with eating and washing, is often provided by relatives rather than nursing staff (Sibai and Yamout 2012). In some of the wealthier Gulf States, foreign live-in domestic workers offer a ‘solution’ to elder care, albeit unregulated posing a number of safeguarding issues (Shah et al. 2012).

Another important factor affecting the role of women in the region is the shift in family formation and structure. Trends toward higher median age at first marriage, urbanization and a decline in co-residence with in-laws and extended families, as well as recent trends towards higher divorce rates, are observable in many countries (Rashad et al. 2005). Currently, most Arab countries are going through some sort of a ‘nuptiality transition’ from one pattern of marriage to another, and different countries are at different stages of this transition. Some observe that the universality of marriage, which characterized the region for many decades, is starting to decline. It is observed that the percentage of women age 20–24 ever married declined from 21 to 14.9 % from the 1980s to 2000 in the region (Hussein 2002; El Haddad 2006; El-Saadani 2006; Mensch et al. 2005; Rashad et al. 2005). These interacting factors call for the consideration of alternative long-term care approaches, including strategies aimed at reducing possible burden on women (traditional informal carers) through support and respite care services, for example. Such burdens may lead to poor health outcomes for all concerned and possible crises requiring expensive health care input.

## Policy Implications

There are several policy implications that arise from the preceding discussion: firstly, what is to be done about the social care for elderly people in the Arab region? Secondly, how might governments support women who have care responsibility for older relatives in the context of competing demands?

Several governments in the region, such as Egypt, Bahrain, Lebanon, Tunisia, Morocco, and Kuwait, have attempted to make conditions easier for working women with the introduction of paid maternity leave; these may be used indirectly to also care for older parents. However, state support for women who provide long-term care for family members, as those present in some developed countries, such as Italy and Spain (Commas-Herrera et al. 2006; Pavolini and Ranci 2008), does not exist. Indeed, the very nature of elderly care being characterized by ‘crisis’ management and not necessarily following a predictable pattern like child care, makes it difficult for women to anticipate when demand is likely to increase.

While there are several social policy legislations in the region that address the needs and wellbeing of the family institution within the society, there are very few that are specific to long term care. For example, most pension programs in the region consider additional benefits if the pensioner has surviving parents. Nevertheless, some countries in the region have attempted to upgrade, or consider, legislation and practical measures that address the growing long term care needs of their elderly populations: for example, the eligibility of elderly care activities to receive tax incentives in Jordan; expanding welfare provision to cover old age disability in Kuwait; initiating long term care insurance provisions in Egypt, Jordan and Oman; considering new pension laws in Lebanon; and facilitating the establishment of long term care day centres and community provisions in Egypt, Jordan and Lebanon (Mirkin 2010). Other innovative practices include establishing mobile units that provide health and care services to older people in Saudi Arabia, Bahrain and Oman.

While these initiatives constitute notable progress, Arab governments still face growing demands to establish sustainable formal long-term care provision as well as to facilitate and support individuals who provide informal care for elderly and disabled family members (Sinunu et al. 2009). To advance this argument further, it is worth considering strategies in more economically developed countries, and other less economically developed countries, as models that can be adapted in the Arab region to address long term care needs. One strand of policies relates to increasing access to care facilities, such as the availability of services including community based care. Very little information is available on the levels or use of care homes or long-term nursing or social care in the region, with studies limited mainly to Egypt, Kuwait, Lebanon and more recently Jordan (Sibai et al. 2004; Sinunu et al. 2009). In a number of the Arab countries, including Egypt and Tunisia, some universities and voluntary sector initiatives play an important role in providing basic health and care services, usually staffed by students and volunteers, to poorer older people (UNFPA 2012). These might be expanded and encouraged by policies or offered financial incentives in the form of tax relief or other benefits.

Another set of policies might focus on facilitating, or working with, informal family support. Some of these approaches address, and seek to improve, work-life balance in general, but rarely have a clear focus on care of older people although there may be a positive impact on informal caregivers. These might be of particular importance, given the multiple roles faced by women and their increased labour participations.

In terms of general work-life strategies, three major types of approaches emerge from the literature in the developed world. First, some countries such as the United Kingdom (UK), New Zealand and Australia have developed ***campaigns to promote work-life balance*** in the workplace by targeting employers. The second can be found in the Netherlands, Sweden and Denmark, where efforts to support people balancing work and other responsibilities, including caring for older people, have been developed through ***a broad range of measures***. The third group, such as France, Belgium, United States and Ireland, focus on ***developing legislation and initiatives*** supporting work-life balance (Hussein et al. 2009). In the first group, a business case is usually presented to promote the value of work-life balance. Employers are encouraged to recognize that access to coping mechanisms will increase workers’ productivity. Initiatives are usually based on providing information on websites or newsletters on the importance of improving work-life balance.

Other policies relate to offering financial assistance to informal carers through tax relief and other allowances. For example, the United Kingdom provides a cash benefit known as Carer’s Allowance, which is means tested based on level of income and employment level of carer. Similarly informal carers in Australia, Germany, Norway and Sweden can draw on a cash allowance, which they can use to purchase formal care if they wished.

The literature highlights the importance of flexible and supportive work environments in enabling people who provide informal care to participate in the labour market (Phillips et al. 2002; Seddon et al. 2004). Although there is some evidence that competing demands may cause carers to cease work completely in favour of their caring responsibilities (Arber and Ginn 1995), the literature also suggests that the majority of carers manage (or are forced to manage) a combination of the two activities (Glendinning 1992; Joshi 1995). Facilitating and supporting informal care is one side of the equation, most of the countries in the Arab region are faced with a pressing need to complement informal care provision if it they are not to be forced to establish more formal long term care systems or to allow it to flourish in an unregulated and unmanaged market (WHO 2000).

## Conclusion

The evidence suggests a growing proportion of older people in the populations of the Arab region. Within a context of changing family structure, dynamic migration patterns and associated risks of ill health in later life, the needs for long-term care support are expanding. The norms, religious and cultural traditions of these countries place the duty of care on women; such care, in the vast majority of cases, is unpaid and informally provided thereby heightening the pressure on women’s wellbeing, time and energy through a combination of competing demands. To offset the impact of the demographic shift and other changes on the traditional system, policymakers in the region must invest in the systems that would encourage and facilitate formal care provision, through partnership between the state and civil society for example and through investing in both old age and family support policies. There is a particular need to foster a regulated and well-organised home-based care services, this would facilitate caring for older people while remaining within their own families and communities. The private sector in Lebanon has pioneered home care services, and two agencies provide home-based nursing care, physiotherapy and palliative care. The review undertaken for this paper highlights the role of civil society in advocacy as well as provision of care within the community, programmes directed towards building capacity should be fostered and subsidised by Governments.

Long term care provision within the region is likely to remain a ‘family business’ for some time to come, thus an approach based on social capital would be most suitable. Such approach would capitalize on intergenerational support and family solidarity through programmes that both empower and support informal care givers, particularly women. How women balance their multiple roles depends on different factors, some operate at the individual level and others relate to the surrounding community and underlying support from government policies. The availability of formal care, within the home environment or the community and whether on a full-time or flexible basis, needs to be considered by almost all countries in the Arab region.

This article argues that governments need to consider policies for both supporting informal care as well as establishing formal long term care provision. One of the ways to approach this process is to ameliorate the competing demands on women and other informal care providers through introducing support measures. Unlike the care of children, which follows a fairly predictable time schedule, care for older or disabled people is unpredictable in duration and intensity; it may also increase in intensity over the course of the care trajectory.

A variety of support measures should be introduced and evaluated, including flexible care arrangements between the formal and informal sector, such as respite care provision. These measures need to be both cost-effective and acceptable through being culturally sensitive. Flexible work environments and the availability of supportive workplace policies would seem to be crucial to enhancing women’s participation in the labour force while not placing undue demands and placing their participation in jeopardy.

Suitable policies need to be tailored and adapted to the cultural context of the region. Policies enabling more flexible working hours and provision of paid or unpaid leave might be particularly important. The availability of informal support to women to ‘care share’ through enlisting the support of partners, older children, friends or other members of the community is also fundamental in facilitating women’s participation in the labour force.

The dual impact of informal care and labour force participation is becoming increasingly important, owing to several factors, including the reality of increased demand for both of them. A large volume of empirical research indicates the difficulties associated with combining care and work responsibilities, particularly caring for older people. While the opportunity exists for countries in the region to adopt and learn from the policy successes and failures of more economically developed countries, this has yet to be implemented.

## Notes

i. Includes the 22 member countries of the League of Arab States: Algeria, Bahrain, Comoros, Djibouti, Egypt, Iraq, Jordan, Kuwait, Lebanon, Libya, Mauritania, Morocco, Oman, Palestine, Qatar, Saudi Arabia, Somalia, Sudan, Syria, the United Arab Emirates, Tunisia and Yemen.
ii. The Human Development Index (HDI) is a summary measure of average achievement in key dimensions of human development: a long and healthy life, being knowledgeable and have a decent standard of living. The HDI is the geometric mean of normalized indices for each of the three dimensions.
iii. Gender Inequality Index measures the human development costs of gender inequality, thus the higher the GII value the more disparities between females and males. The GII values vary tremendously across countries, they range from 2.1 % to 73.3 %. Source: World Economic Forum, The Global Gender Gap Report 2010- http://hdr.undp.org/en/data
